# Exploring gene-drug interactions for personalized treatment of post-traumatic stress disorder

**DOI:** 10.3389/fncom.2023.1307523

**Published:** 2024-01-11

**Authors:** Konstantina Skolariki, Panagiotis Vlamos

**Affiliations:** Bioinformatics and Human Electrophysiology Laboratory, Department of Informatics, Ionian University, Corfu, Greece

**Keywords:** post-traumatic stress disorder, genetic biomarkers, mathematical modeling, system pharmacology, functional analysis

## Abstract

**Introduction:**

Post-Traumatic Stress Disorder (PTSD) is a mental disorder that can develop after experiencing traumatic events. The aim of this work is to explore the role of genes and genetic variations in the development and progression of PTSD.

**Methods:**

Through three methodological approaches, 122 genes and 184 Single Nucleotide Polymorphisms (SNPs) associated with PTSD were compiled into a single gene repository for PTSD. Using PharmGKB and DrugTargetor, 323 drug candidates were identified to target these 122 genes. The top 17 drug candidates were selected based on the statistical significance of the genetic associations, and their promiscuity (number of associated genestargets) and were further assessed for their suitability in terms of bioavailability and drug-like characteristics. Through functional analysis, insights were gained into the biological processes, cellular components, and molecular functions involved in PTSD. This formed the foundation for the next aspect of this study which was to propose an efficient treatment for PTSD by exploring drug repurposing methods.

**Results:**

The main aim was to identify the drugs with the most favorable profile that can be used as a pharmacological approach for PTSD treatment. More in particular, according to the genetic variations present in each individual, the relevant biological pathway can be identified, and the drug candidate proposed will specifically target said pathway, accounting for the personalized aspect of this work. The results showed that the drugs used as off-label treatment for PTSD have favorable pharmacokinetic profiles and the potential drug candidates that arose from DrugTargetor were not very promising. Clozapine showed a promising pharmacokinetic profile and has been linked with decreased psychiatric symptoms. Ambrucin also showed a promising pharmacokinetic profile but has been mostly linked with cancer treatment.

## 1 Introduction

The 5^th^ edition of the Diagnostic and Statistical Manual of Mental Disorders (DSM-5) identifies Post-Traumatic Stress Disorder (PTSD) as an anxiety disorder that can be developed after an individual is exposed to a traumatic event that involves actual or threatened death, serious bodily harm, or sexual violation. This exposure consequently results in feelings of fear, helplessness, or horror (Pai et al., 2017). PTSD is a multifactorial disorder affected by various factors (i.e., demographic, biological, environmental, psychological, as well as social). In terms of neurobiological aspects, several biological pathways have been shown to contribute to the development of PTSD. These pathways include but are not limited to, synaptic transmission, neuroinflammation, the hypothalamic–pituitary–adrenal (HPA) axis, metabolic processes, and neuroendocrine signaling have all been identified as important factors. Research has shown that by targeting synaptic transmission, and specifically by inhibiting the Hyperpolarization Activated Cyclic Nucleotide Gated Potassium Channel 1 (HCN1) channels involved in electrical activity, PTSD symptoms can be reduced (Ni et al., 2020). Additionally, other studies have highlighted the significance of genes like Brain-derived neurotrophic factor (BDNF) in facilitating synaptic and neurotransmitter transmission.

Dysregulation in certain neurotransmitters such as GABA, glutamate, norepinephrine, dopamine, and serotonin as well as genetic variations in the genes that encode for these neurotransmitters have been implicated with PTSD and in particular in stress response and mood regulation (Bennett et al., 2016). This evidence is also aligned with the results of the analysis shown in the following sections (Murnane, 2019; Ney et al., 2021). Serotonin also plays a role in regulating the HPA axis via its activation through the Serotonin 2C Receptor Stimulation. The HPA regulates stress response predominantly via a cascade of events. Specifically, in response to a stressor, corticotropin-releasing hormone (CRH) is released by the hypothalamus, which leads to the release of adrenocorticotropic hormone (ACTH) by the pituitary gland. As a result, the adrenal cortex is stimulated, and cortisol is secreted. Once cortisol levels rise in the body, they signal the hypothalamus and pituitary gland to stop CRH and ACTH release respectively, effectively completing the feedback loop. In PTSD however, there are certain dysregulations in this cascade of events such as impaired feedback in the negative feedback loop which can result in prolonged stress response. Furthermore, evidence suggests that ACTH levels are higher in PTSD (D’Elia et al., 2021). There has not been consistent evidence on whether the HPA axis is hyper- or hypo-active in PTSD. These inconsistencies can be attributed to factors such as individual differences, comorbidities, as well as the timing of cortisol measurements which can impact the observed activity of the HPA axis. HPA axis is activated by the release of the corticotropin-releasing factor (CRF) usually resulting after exposure to a trigger or a stressor. In PTSD, imbalances in the HPA axis have also been linked to disruptions in CRF levels (Ramos-Cejudo et al., 2021). Furthermore, irregularities in the HPA axis can lead to fluctuations in glucocorticoids (GCs) levels. Alterations in glucocorticoid signaling as well as in glucocorticoid receptors (GR) have also been implicated with PTSD. An enhanced sensitivity of the GR which has been observed in PTSD and which accounts for the increased negative feedback inhibition of cortisol release in the HPA axis, could also be responsible for the alterations in the stress response observed in the disorder (Szeszko et al., 2018). Similar to the contradicting evidence in regard to the hyper- and hypo-activation of the HPA axis, cortisol levels in PTSD also show conflicting findings. However, in contrast to acute stress response, PTSD can be characterized as a persistent response to a stressor that is no longer present and lower cortisol levels are more representative of stress response dysregulations (Daskalakis et al., 2016). More in particular, in PTSD cases, increased CRH as a response to stress, with reduced ACTH levels which consequently lead to reduced cortisol levels have been observed. As a result, the hypothalamus perceives this and increases the release of CRH with the aim of increasing cortisol. However, due to dysregulation, the ACTH levels do not increase and therefore the cortisol levels are not sufficient to ensure homeostasis. This is further supported by the exaggerated effect of the negative feedback loop. HPA dysregulation also leads to decreased GC signaling and consequent increased GR sensitivity. This increased GR sensitivity can also be associated with the increased negative feedback loop. When GR sensitivity is increased, the cells are more responsive to glucocorticoids. The dysregulation of the HPA axis and imbalances in stress hormones as well as subsequent imbalances in brain chemicals can contribute to the development of PTSD and the persistence of symptoms like heightened alertness, avoidance, and reliving traumatic experiences (Dunlop and Wong, 2019). Similarly to serotonin dysregulation, disruptions in norepinephrine levels, another neurotransmitter associated with the “fight or flight” response, have been connected to hyperarousal symptoms experienced by individuals with PTSD (Hendrickson et al., 2018). Elevated norepinephrine levels have been observed in people with PTSD, contributing to their heightened state of arousal.

Other monoamine neurotransmitters implicated in PTSD include dopamine. Dopamine deficiency, altered dopamine transmission (Enman, 2015) as well as variation in the genes encoding dopamine receptors (e.g., DRD2, DRD3) have been implicated with PTSD and increased PTSD susceptibility (Wolf et al., 2014). For many if not all of the aforementioned biological and molecular drivers in regard to PTSD, there is a hidden gene component involved. For example, in the case of HPA dysregulation, FKBP5 genetic variations can be considered accountable for said imbalances via decreased negative feedback between FKBP5, GRs, and GCs, as well as the dysregulation of GR sensitivity (Malekpour et al., 2023). Epigenetic changes such as FKBP5 methylation have also been associated with HPA imbalances and PTSD symptoms (Grasso et al., 2020). Genetic variations in the GR gene (NR3C1) have also been implicated in the consequent HPA axis dysregulations (Daskalakis et al., 2016).

Understanding the aforementioned mechanisms is crucial for developing effective treatment strategies for PTSD (Banerjee et al., 2017). By compiling a repository of genes and related Single Nucleotide Polymorphisms (SNPs), the pharmacological methodology can have a gene-based focus which not only adds a personalized aspect but also appears more promising in terms of effectiveness. Currently, only sertraline and paroxetine have gained FDA approval for the treatment of PTSD (Ehret, 2019). These two drugs belong to a category known as Selective Serotonin Reuptake Inhibitors (SSRIs). SSRIs and Serotonin-Norepinephrine Reuptake Inhibitors (SNRIs) are first-line pharmacological interventions for PTSD (Bernardy and Friedman, 2015). As their name suggests, these antidepressants work by modulating the levels of neurotransmitters such as serotonin and norepinephrine, which are implicated in mood regulation, anxiety, and stress responses. Even though they have proven to be efficient in alleviating certain PTSD symptoms, including intrusive thoughts, hyperarousal, and avoidance behavior (Huang et al., 2020), their effectiveness is improved when combined with psychotherapy. The main aim of this work is to identify potential drug candidates for repurposing in PTSD using a gene-driven approach for higher accuracy as well as the development of a mathematical model to further support drug repurposing approaches.

## 2 Methodology

### 2.1 Identification of PTSD-associated genes

**Figure 1:**
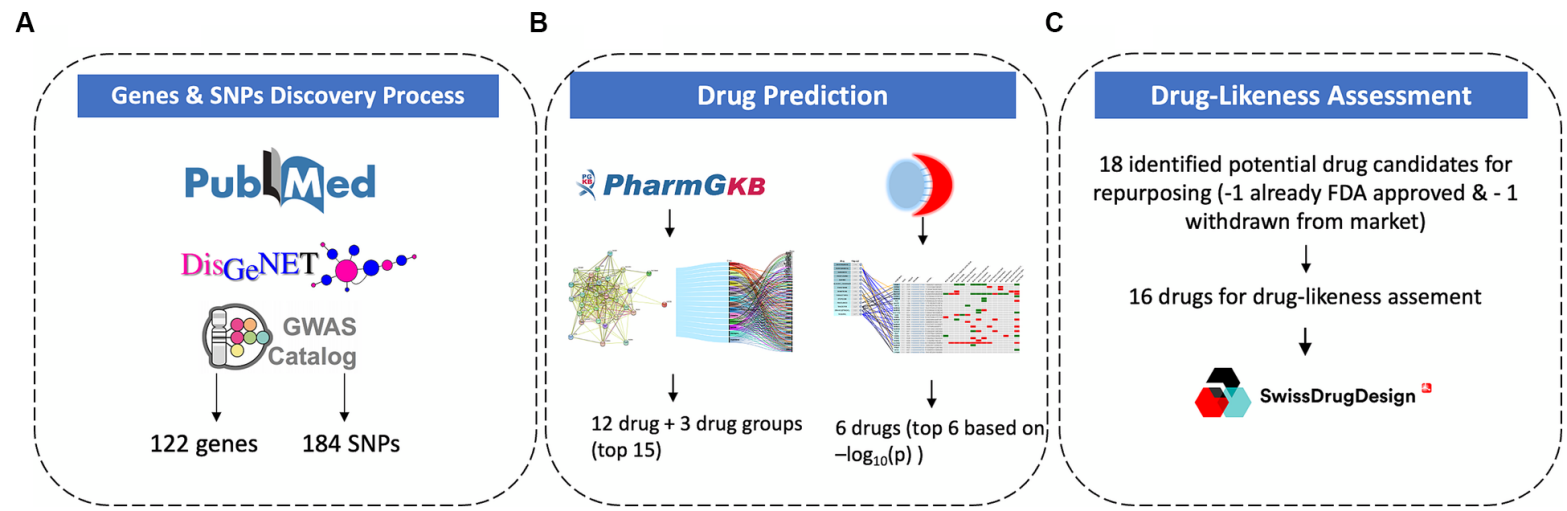
Methodology for gene and SNPs discovery process **(A)**, drug prediction **(B)**, and drug-likeness assessment **(C)**.

The study focused on identifying the genes with corresponding known or suspected SNPs associated with PTSD. This targeted approach increases the likelihood of studying genes that are directly involved in the mechanisms associated with the disease. Furthermore, the aim of this study is to identify effective drug candidates for repurposing in PTSD. Therefore, SNPs were chosen as a primary focus in order to identify disease-causing genes and understand inter-individual variations in drug response. SNPs represent specific points of variation within genes which allows pinpointing of precise genetic differences that may influence traits, disease, or drug responses. The data collection process (Figure 1A) was based on three axes: 1. Literature mining from PubMed, 2. Computational mining for genes via DisGeNET, and 3. GWAS identification via the GWAS Catalogue.

#### 2.1.1 PubMed: literature mining

A systematic literature search was conducted on PubMed with the query “Post-Traumatic Stress Disorder” OR “PTSD” AND “genes” OR “genetic variations.” Research articles, clinical trials, randomized controlled trials, and meta-analyses, from 2010 until June 2023, focusing on the genetic factors associated with PTSD were retrieved. Information regarding candidate genes, genetic variants, and their potential role in PTSD development was extracted from the 41 resulting papers. From these papers 13 were excluded seeing as 6 were not relevant, 6 did not provide specific variations and 1 was older than 2010 (publication date 2005). From these 28 final papers, 61 SNPs were extracted.

#### 2.1.2 DisGeNET: identification of PTSD-associated genes

The DisGeNET database^1^ was accessed to identify genes associated with PTSD. Using the medical subject heading (MeSH) term “Post-Traumatic Stress Disorder” (MeSH ID: C0038436), a query was performed. The retrieved data included gene-disease associations, genetic variations, and supporting references. The query yielded 117 genetic variants. The duplicates between the PubMed mining and DisGeNET mining were removed. To ensure that the genetic variants were correctly identified as relevant to PTSD, the corresponding citations were searched using the keyword “PTSD” or “Post-traumatic stress disorder” or “posttraumatic stress disorder” in the DisGeNET database and using the associated resulting PubMed ID (PMID) the research at hand was identified. From the 117 genetic variants provided from the query, 5 SNPs were not included in the results seeing as the corresponding publication showed no significant association with PTSD, 3 SNPs were not included seeing as they originated from a study before 2010 and the results were not replicated thereafter, 3 SNPs were removed seeing as there were no corresponding citations for validation purposes.

#### 2.1.3 GWAS catalogue: identification of PTSD-associated genetic variants

To ensure all the GWAS studies related to PTSD have been accounted for, the GWAS Catalogue^2^ was accessed and the trait search term “post-traumatic stress disorder” was used to perform a search. The results provided a list of GWAS studies associated with PTSD. From there, all studies were examined and the ones that provided information on significant genetic variants linked to PTSD were included in the results. From the 138 unique associations yielded in the search, 78 were removed seeing as they were not validated from any corresponding study and 60 were included in the results. After combining the results from these 3 methodological (Figure 1A) approaches and removing duplicate genes and SNPs, the final PTSD-related gene set included 122 genes and 184 SNPs.

### 2.2 Methodology for drug-target interactions

The gene set of 122 genes identified as related to PTSD based on the aforementioned methodology was uploaded into GeneCodis and the Drug analysis for Pharmacogenomics Knowledge Base (PharmGKB)^3^ was performed. This analysis provided a list of 309 drugs that target the candidate genes associated with PTSD. Each interaction represented a potential drug-gene association that may be relevant for PTSD treatment. Based on the number of gene targets, some drugs were considered more promiscuous than others.

### 2.3 Methodology for genetics-driven drug-target network

DrugTargetor^4^ was used to identify drugs and genes that are arranged by GWAS-derived scores. The drug score is −log10(*p*-value) as calculated from the drug/phenotype association test, which was performed using MAGMA pathway analysis. The gene scores of 1–7 were calculated using the MAGMA gene-wise association test and S-PrediXcan. The highest score (7) indicated that the gene was significant in both calculations. For this study, the largest GWAS of PTSD (*N* = 20.070) (Duncan et al., 2018) was used. Furthermore, in order to avoid network connections derived from text mining approaches, and to retain more reliable connections the ChEMBL/KiDB bioactivities were chosen as a connection type.

### 2.4 Methodology for enrichment analysis and protein–protein interaction network

For the genes identified as targets for the top drugs, an enrichment analysis was performed using g:Profiler^5^ (Kolberg et al., 2023). The query was filtered to only provide results for “*Homo sapiens*”, and the following Gene Ontology (GO) Enrichment Analyses were compiled; Biological Process (BP) which identifies overrepresented biological processes that the genes are involved in, Molecular Function (MF) which shows the enriched molecular functions associated with the genes and Cellular Component (CC) which indicates enriched cellular components in which the genes are involved in. Network plots for the genes involved in GO:BP, GO:MF, and GO:CC were additionally analyzed and visualized using GeneCodis^6^ (Garcia-Moreno et al., 2022). The KEGG Pathway Analysis was performed using the “clusterProfiler” package (17) in R software (version 4.3.1).^7^ An additional KEGG Pathway analysis was performed using ShinyGO v0.51.^8^ The parameters were set at *p* = 0.05 cutoff and *q* = 0.05 cutoff. The Protein–Protein Interaction (PPI) Network Analysis that created the PPI network based on known interactions among the uploaded genes was performed using the Search Tool for the Retrieval of Interacting Genes (STRING v.12).^9^ STRING identifies interconnected protein clusters within the network and predicts the functional interactions of proteins (Szklarczyk et al., 2023). STRING was also used to identify gene co-expression to predict and recognize any potential functional associations. A PPI analysis was also performed using the Metascape tool^10^ (Zhou et al., 2019).

### 2.5 Drug-likeness assessment

Drug-likeness assessment using SwissADME^11^ was conducted to evaluate the potential of molecules for absorption, distribution, metabolism, and excretion (ADME), physicochemistry, pharmacokinetics, and medicinal chemistry friendliness properties (Figure 1C). This methodology was applied to the top 17 drug candidates identified using the methodologies described above (Figure 1B). The Drug-likeness section of SwissADME offers five rule-based filters, derived from analyses by major pharmaceutical companies, to determine drug-likeness based on different property ranges. SwissADME’s drug-likeness assessments aim to provide rapid screenings of chemical libraries, aiding in the selection of molecules for further development stages. The predictions help identify compounds that align with specific project-related demands and chemical requirements.

## 3 Results

### 3.1 Gene-based potential repurposing candidates

**Figure 2:**
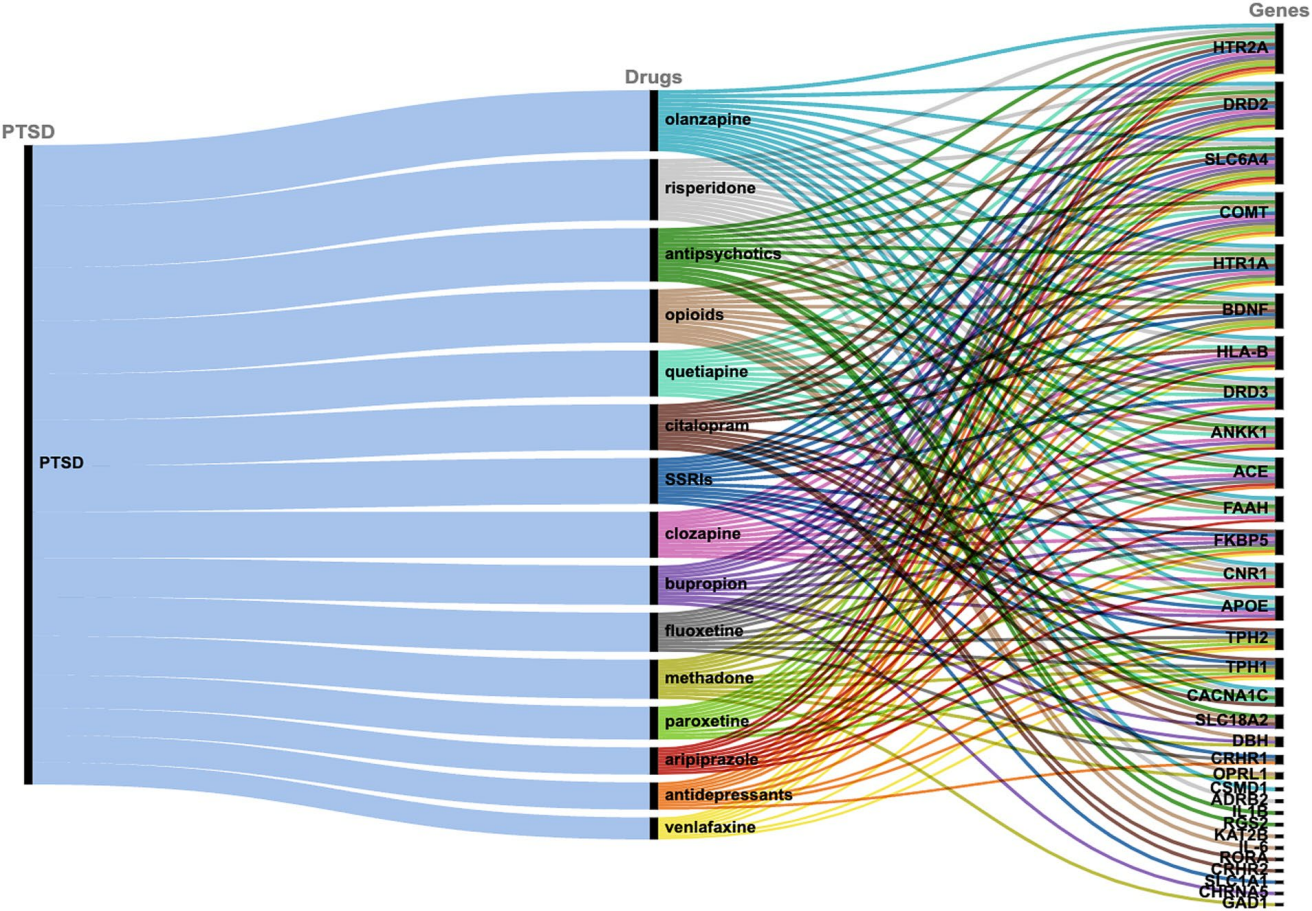
Alluvial diagram showing the correlation between PTSD, the top 15 drugs as identified via PharmGKB, and the associated target genes.

The drug-target interactions associated with PTSD were investigated based on the PTSD-related gene set identified (*n* = 122 genes). The analysis yielded a comprehensive network that provides insights into the connections between the drugs and the targeted genes implicated in the pathophysiology of PTSD. Each drug is linked to a specific set of genes, indicating potential interactions that may influence the treatment response and efficacy in individuals affected by PTSD. In particular, 309 drugs were identified that exhibited associations with multiple genes (or in some cases one or a few genes), suggesting potential polygenic effects in the context of treatment response and therapeutic outcomes for PTSD. To assess the significance of the drug-target interactions, an enrichment analysis was performed. Among the drugs investigated, certain ones exhibited statistically significant enrichment for their associations with PTSD-related genes, indicating potential therapeutic relevance. The top 15 drugs include quetiapine, venlafaxine, fluoxetine, bupropion, olanzapine, citalopram, antipsychotics, paroxetine, risperidone, opioids, methadone, aripiprazole, SSRIs, clozapine, and antidepressants which showed highly significant associations with PTSD-related genes (Figure 2, Table 1). These findings highlight the importance of further research to explore the mechanisms of action and clinical implications of these drug-target interactions in the context of PTSD treatment.

**Table 1:** PharmGKB genes-target of the top 15 predicted drugs.

Drugs	pval	pval_adj	Genes
Olanzapine	1,01E+07	6,22E+07	CSMD1, FAAH, HLA-B, HTR1A, BDNF, APOE, DRD3, CNR1, ACE, CACNA1C, HTR2A, SLC6A4, DRD2, COMT, ANKK1
Risperidone	5,81E+07	2,24E+09	ADRB2, FAAH, HLA-B, HTR1A, BDNF, APOE, DRD3, CNR1, ACE, CACNA1C, HTR2A, SLC6A4, DRD2, COMT, ANKK1
Antipsychotics	1,63E+06	8,41E+07	IL1B, FAAH, HTR1A, BDNF, DRD3, ACE, CACNA1C, HTR2A, SLC6A4, SLC18A2, RGS2, DRD2, COMT, ANKK1
Opioids	1,07E+08	3,18E+08	FAAH, HTR1A, BDNF, KAT2B, OPRL1, DBH, DRD3, CNR1, HTR2A, IL-6, SLC18A2, DRD2, COMT, ANKK1
Quetiapine	1,40E+05	2,40E+07	FAAH, HLA-B, HTR1A, APOE, DRD3, CNR1, ACE, CACNA1C, HTR2A, SLC6A4, DRD2, COMT, ANKK1
Citalopram	8,38E+05	6,22E+07	TPH2, HLA-B, HTR1A, FKBP5, BDNF, RORA, TPH1, CACNA1C, HTR2A, CRHR2, SLC6A4, SLC18A2, DRD2
Selective serotonin reuptake inhibitors	1,84E+08	4,73E+09	TPH2, CRHR1, HTR1A, FKBP5, SLC1A1, BDNF, APOE, DRD3, TPH1, HTR2A, SLC6A4, DRD2, COMT
Clozapine	3,35E+07	7,97E+08	FAAH, HLA-B, HTR1A, FKBP5, APOE, DRD3, CNR1, ACE, HTR2A, SLC6A4, DRD2, COMT, ANKK1
Bupropion	1,61E+06	2,40E+07	HLA-B, FKBP5, DBH, APOE, ACE, CHRNA5, HTR2A, SLC6A4, SLC18A2, DRD2, COMT, ANKK1
Fluoxetine	2,33E+05	2,40E+07	TPH2, HLA-B, CRHR1, HTR1A, FKBP5, BDNF, ACE, TPH1, HTR2A, SLC6A4, DRD2, COMT
Methadone	1,13E+08	3,18E+08	TPH2, GAD1, BDNF, OPRL1, DBH, CNR1, TPH1, HTR2A, SLC6A4, DRD2, COMT, ANKK1
Paroxetine	5,74E+06	2,24E+09	TPH2, HLA-B, HTR1A, FKBP5, BDNF, DRD3, TPH1, HTR2A, SLC6A4, DRD2, COMT
Aripiprazole	9,49E+05	3,18E+08	FAAH, HLA-B, APOE, DRD3, CNR1, ACE, HTR2A, SLC6A4, DRD2, ANKK1
Antidepressants	6,12E+08	9,45E+09	TPH2, CRHR1, HTR1A, FKBP5, BDNF, ACE, TPH1, HTR2A, SLC6A4, COMT
Venlafaxine	1,61E+09	3,11E+10	TPH2, HLA-B, HTR1A, FKBP5, TPH1, HTR2A, SLC6A4, DRD2, COMT

### 3.2 Disease-based potential repurposing candidates

**Figure 3:**
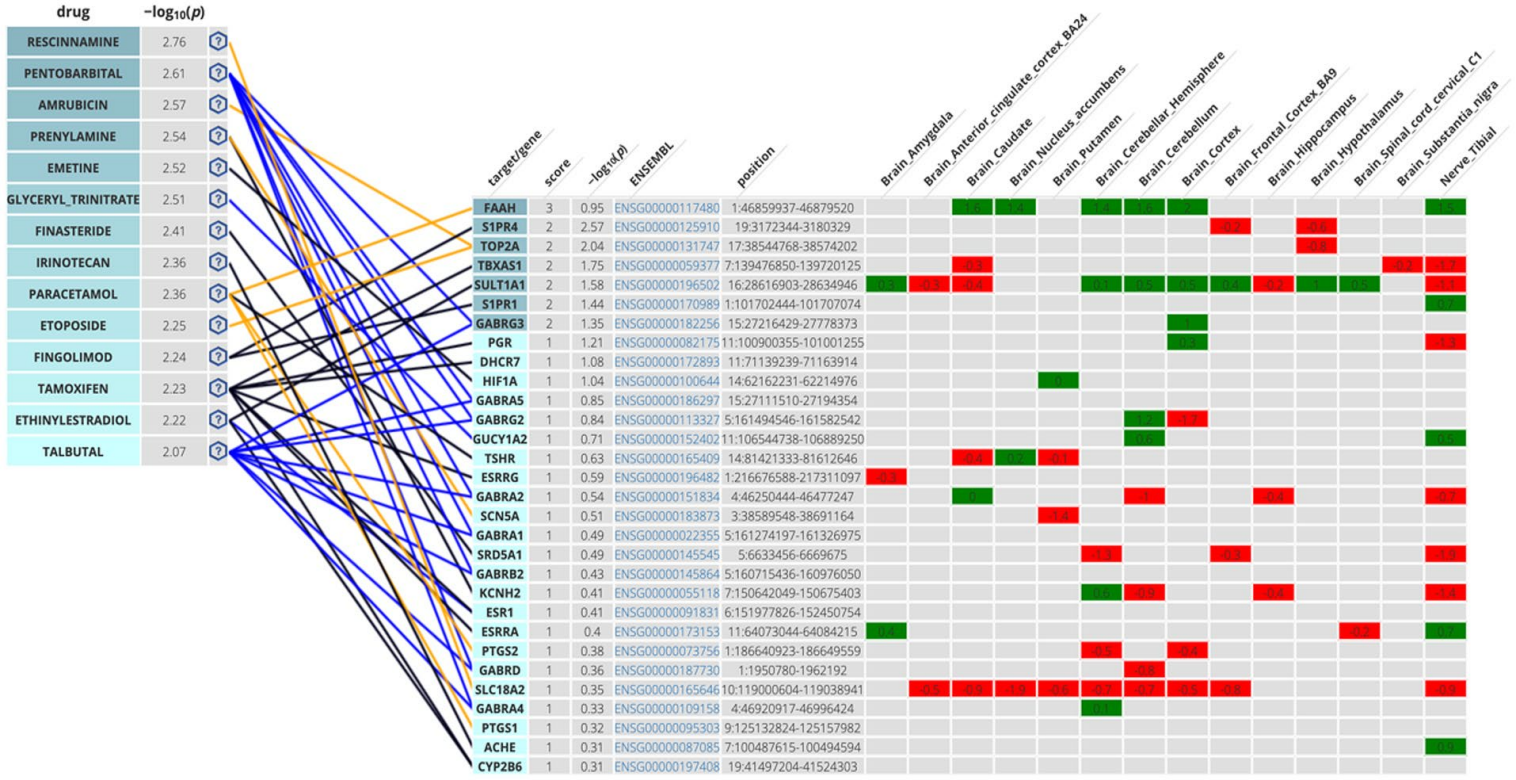
Genetics-Driven Drug-Target Network that shows drug and target associations. The following color code was adopted in the target table for S-PrediXan associations: green = predicted increased expression, red = predicted decreased expression. The blue lines indicate an agonist/activator/positive modulator network connection, the yellow lines indicate an antagonist/inhibitor/negative modulator network connection, and the black lines undefined bioactivity.

The second methodological approach for identifying potential compounds for repurposing using DrugTargetor, showed that the drugs with the higher scores include Rescinnamine, Pentobarbital, Amrubicin, Prenylamine, Emetine, and Glyceryl Trinitrate (Figure 3). These drugs represent excellent candidates for further investigation, given their potential for targeted therapies and fewer off-target effects. Rescinnamine is shown to act as an antagonist/ negative modulator to SLC18A2, a gene with the lowest gene score (1). The Solute carrier family 18 member A2 (SLC18A2) gene encodes the vesicular monoamine transporter 2 (VMAT2) protein, which is involved in packaging and transporting monoamine neurotransmitters. Several polymorphisms in the SLC18A2 gene have been found in relation to PTSD, with the top SNP being rs363276 (Solovieff et al., 2014), which has been associated with increased risk for PTSD (Bharadwaj et al., 2018). Pentobarbital acts on GABRG3, GABRG5, GABRG2, GABRA2, GABRA1, GABRB2, GABRD and GABRA4 as an agonist/ positive modulator. All these genes except for GABRG3 (which has a gene score of 2) have a gene score of 1. These genes encode for subunits of the GABAA receptors, an inhibitory type of neurotransmitter. Amrubicin targets TOP2A as an antagonist/ negative modulator. TOP2A has one the highest scores among the genes of this analysis (gene score of 2). This gene also showed the second highest association with PTSD (−log10(p) 2.04). TOP2A encodes an enzyme, a DNA topoisomerase, that regulates and modifies the topologic states of DNA during transcription. Prenylamine targets SCN5A (gene score of 1) as an antagonist/ negative modulator and KCNH2 (unknown mode of action/ gene score of 1). The SCN5A gene encodes an integral membrane protein and tetrodotoxin-resistant voltage-gated sodium channel subunit responsible for the proper function of cardiac muscle cells, playing a fundamental role in the generation and propagation of electrical signals in the heart (Li et al., 2018). The KCNH2 gene encodes a component of a voltage-activated potassium channel engaged in regulating the flow of potassium ions in cardiac muscle cells, nerve cells, and microglia. Emetine targets HIF1A (gene score of 1) via an unknown mode of action. The HIF1A gene encodes the alpha subunit of transcription factor hypoxia-inducible factor-1 (HIF-1) which plays a central role in a central role in the adaptation and control of oxygen homeostasis within cells. Glyceryl Trinitrate targets GUCY1A2 (gene score of 1) as an agonist/ positive modulator. GUCY1A2 encodes an alpha subunit of the guanylate cyclase enzyme which influences various physiological processes such as neurotransmission. The gene S1PR4 showed the highest association with PTSD (−log10(p) 2.57), followed by TOP2A and TBXAS1 (−log10(p) 1.75), SULT1A1 (−log10(p) 1.58), S1PR1 (−log10(p) 1.44), and GABRG3 (−log10(p) 1.35). S1PR4 is part of the G-protein-coupled (EDG) receptor gene family and is involved in cell signaling and other physiological processes. TBXAS1 a member of the cytochrome P450 superfamily of enzymes and it is involved in several in several pathophysiological processes. SULT1A1 belongs to the sulfotransferase family. S1PR1 encodes a protein structurally similar to G protein-coupled receptors and among others is involved in the regulation of endothelial cells differentiation. This drug-target network analysis allows for the identification of potential associations between drugs and targets based on predicted gene expression changes. This provides a starting point for exploring novel indications for existing drugs.

### 3.3 Enrichment analysis of PTSD predicted drugs’ targets

**Figure 4:**
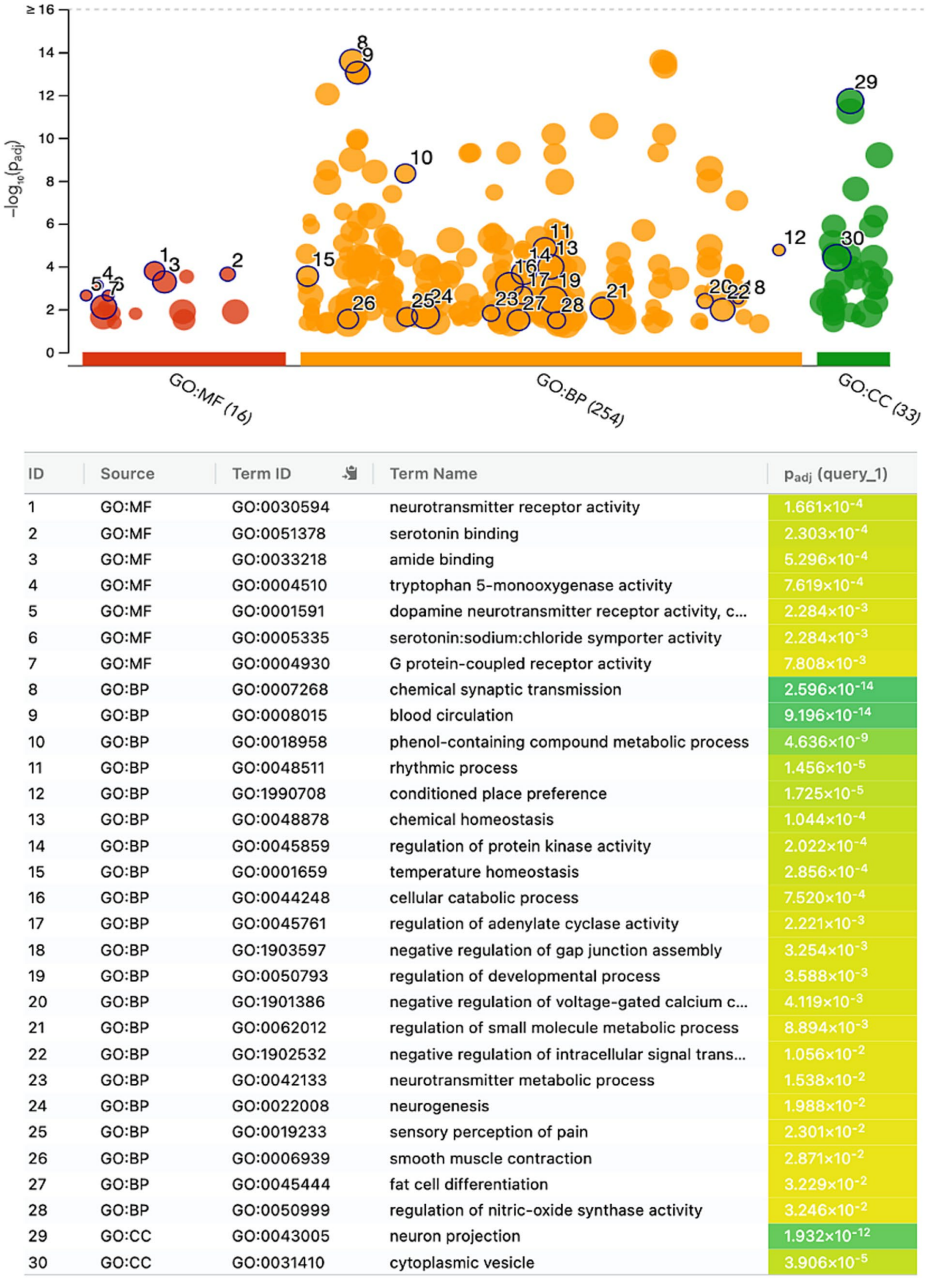
Gene ontology enrichment analysis of the genes-target of the top 17 predicted drugs; Gene Ontology Molecular Function (GO:MF), Gene Ontology Biological Processes (GO:BP), and Gene Ontology Cellular Component (GO:CC).

**Figure 5:**
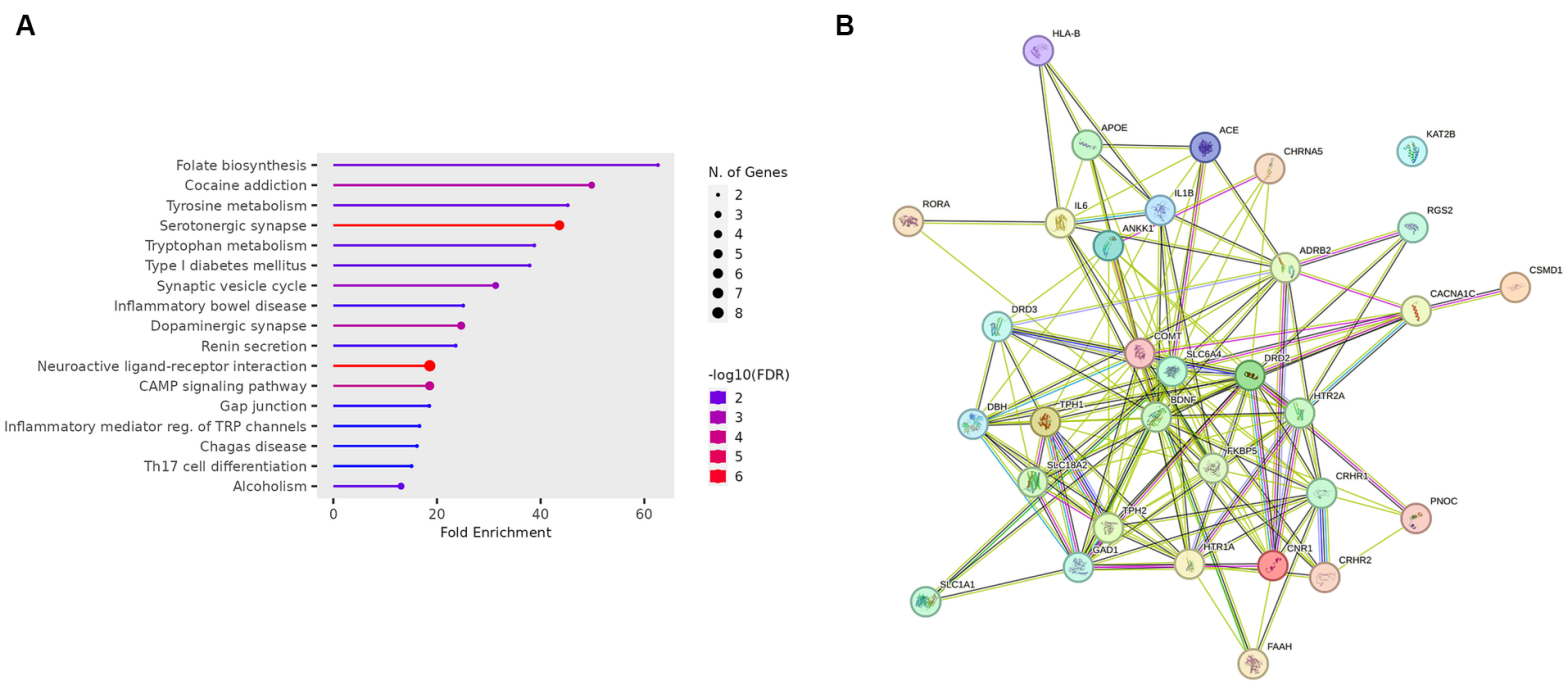
**(A)** KEGG enrichment analysis of the genes-target of the top 17 predicted drugs. **(B)** PPI network of the genes-target of the top 17 predicted drugs.

The gene ontology enrichment analysis conducted on the identified list of genes—pharmacological targets of the top 17 drugs (*n* = 37 unique genes/ *n* = 186 gene targets in total) provided valuable insights into the potential roles of these genes in various biological processes, molecular functions, and cellular components (Figure 4). The molecular functions highlighted (Figure 4), such as neurotransmitter receptor activity and serotonin binding, align with existing knowledge of neurotransmitter dysregulation in PTSD. Other enriched terms included GO:MF “Amide binding” and “Tryptophan 5-monooxygenase activity,” “Chemical synaptic transmission,” “Blood circulation,” “Phenol-containing compound metabolic process,” “Rhythmic process,” “neuron projection,” “cytoplasmic vesicle” (Figure 4). The KEGG analysis also showed the enrichment of the folate biosynthesis pathway (Figure 5A). The pathways associated with “cocaine addiction” and “alcoholism” highlight the intricate relationship between substance use disorders and PTSD. The protein–protein interaction (PPI) network was established using the proteins encoded from the aforementioned 37 genes. This network shows the intricate relationships and potential collaborative roles among the identified genes with other potentially disease-related genes (Figure 5B). The observed number of interactions significantly surpasses the expected number, as indicated by the PPI enrichment *p*-value of <1.0e-16. This enrichment suggests that the genes in the network exhibit more interactions among themselves than what would be anticipated by chance, implying that they are biologically connected in a coordinated manner. Furthermore, the network highlights hub genes that exhibit a higher degree of connectivity, potentially serving as key regulators or mediators of interactions within the system. Depending on the genetic variations present in each patient and utilizing the aforementioned functional analysis, the proposed pharmacological treatment will be chosen in order to target the specific variations and pathways.

### 3.4 Drug evaluation for repurposing

**Figure 6:**
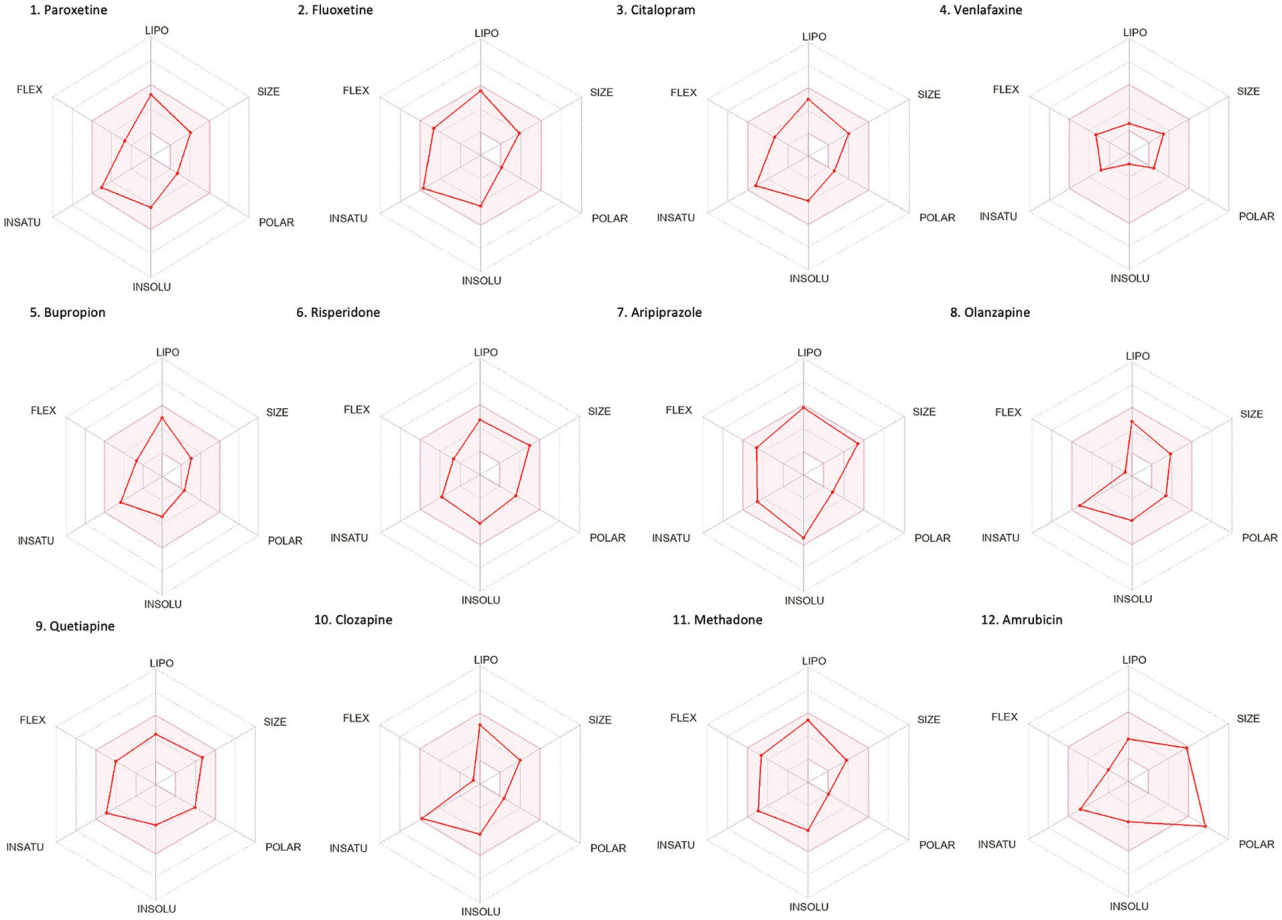
Bioavailability radars of the top drug compounds identified using PharmGKB. The shaded region illustrates the ideal interval for each attribute; lipophilicity (LIPO): XLOGP3 ranging from −0.7 to +5.0, SIZE: MW falling within 150–500 g/mol, polarity (POLAR): TPSA within 20–130 Å2, solubility (INSOLU): log S below 6, saturation (INSATU): fraction of sp3 hybridized carbons not below 0.25, and flexibility (FLEX): limited to a maximum of 9 rotatable bonds. As an example, in part 5. Bupropion: the compound shows high lipophilicity, moderate solubility, relatively low polarity, moderate saturation, flexibility, and size. Based on these, the oral bioavailability of bupropion could be predicted to be moderate to high.

**Figure 7:**
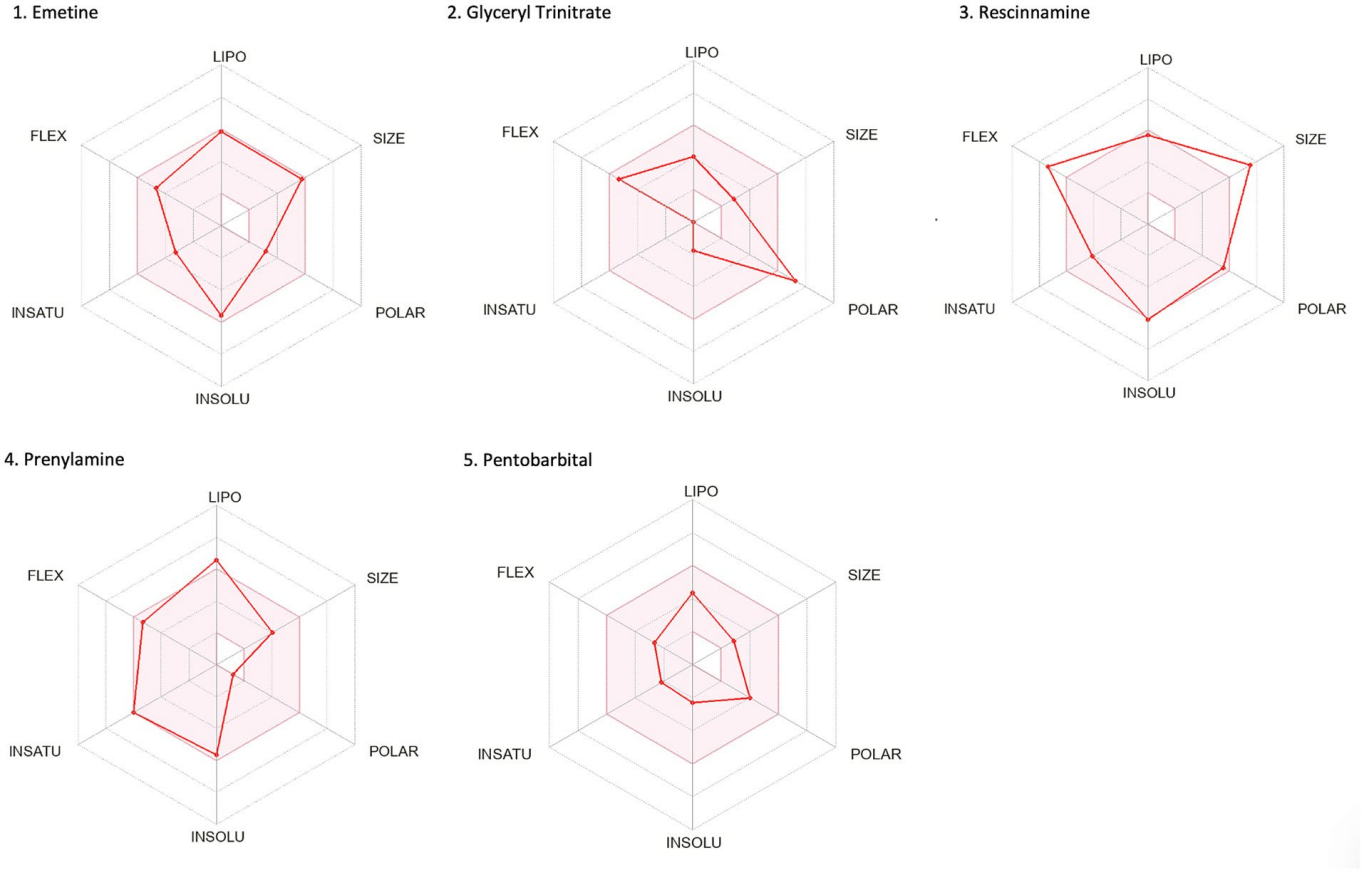
Bioavailability radars of the top drug compounds identified using DrugTargetor. The shaded region illustrates the ideal interval for each attribute; lipophilicity (LIPO): XLOGP3 ranging from −0.7 to +5.0, SIZE: MW falling within 150–500 g/mol, polarity (POLAR): TPSA within 20–130 Å2, solubility (INSOLU): log S below 6, saturation (INSATU): fraction of sp3 hybridized carbons not below 0.25, and flexibility (FLEX): limited to a maximum of 9 rotatable bonds. As an example, in part 3. Rescinnamine: the compound shows moderate lipophilicity, increased size, poor solubility and increased flexibility. Based on these, the oral bioavailability of Rescinnamine is predicted to be low.

**Figure 8:**
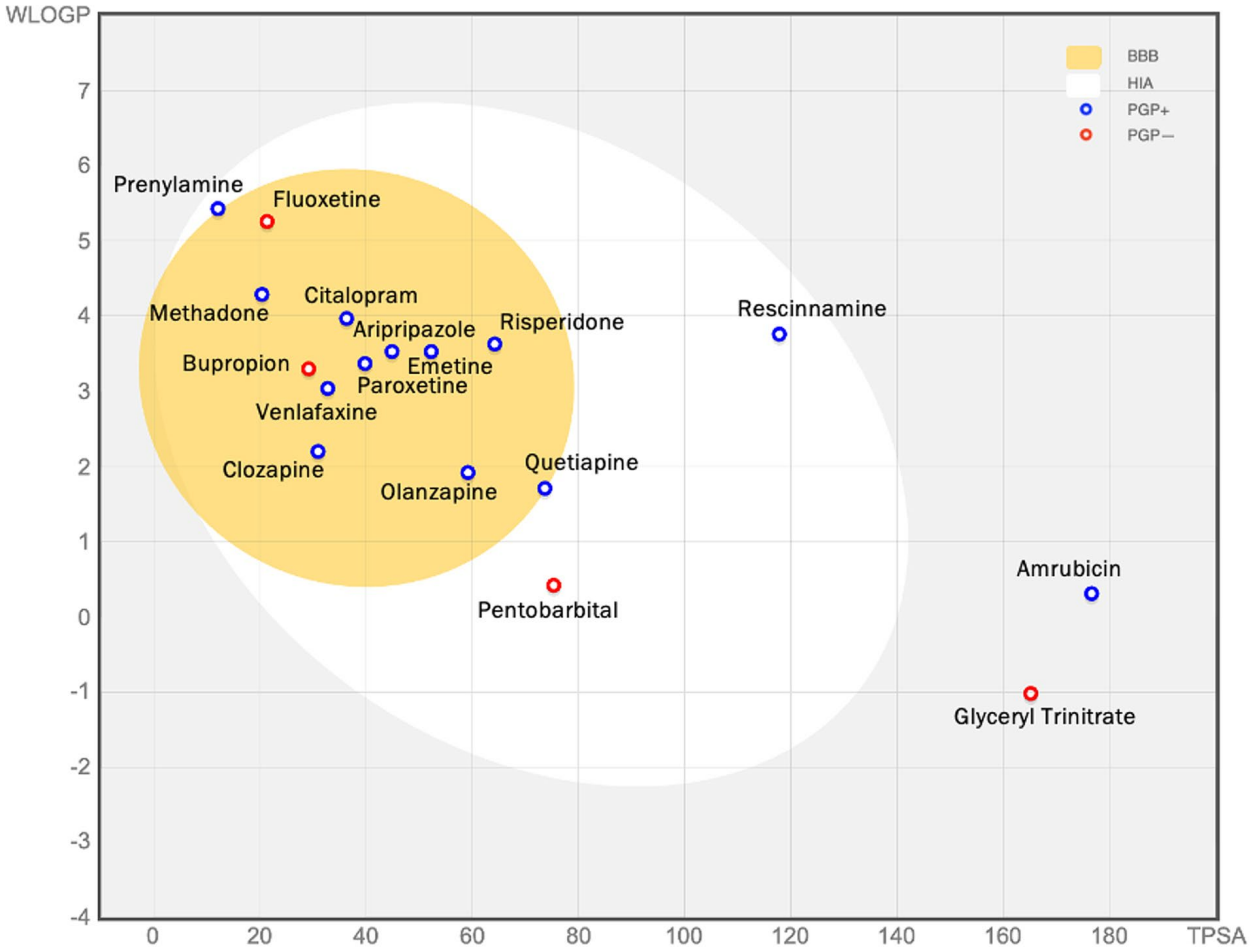
The figure (the BOILED egg) shows the assessment of passive gastrointestinal absorption (HIA) and brain penetration (BBB) based on the positioning of molecules in the WLOGP—versus—TPSA reference plot. The high likelihood of passive absorption by the gastrointestinal tract is represented by the white region, while the yellow (yolk) region indicates a high likelihood of brain penetration. It’s important to note that the yolk and white areas can coexist. Additionally, points are shaded blue if they are predicted to be actively effluxed by P-gp (PGP+), and red if they are predicted to not be substrates of P-gp (PGP−). For example, glyceryl trinitrate is predicted as not absorbed and not brain penetrant (outside the Egg), and aripiprazole is predicted as well-absorbed and it can penetrate the brain (in the white and yellow), and PGP+ (blue dot).

Drug-likeness assessment (Figures 6-8, Table 2) is a process used in drug discovery and development in order to evaluate the potential of a compound (Daina et al., 2017). Figures 6, 7 show a radar chart for each of the previously identified top potential drug candidates for repurposing in the framework of PTSD. The following criteria were used to establish whether the identified potential drug candidates showed favorable characteristics to establish drug viability for further exploration in drug discovery and development processes: chemical structure and bioavailability, physicochemical properties, lipophilicity, water solubility, pharmacokinetics, drug-likeness, and medicinal chemistry. Bupropion, quetiapine, fluoxetine, olanzapine, citalopram, clozapine, venlafaxine, and risperidone demonstrated favorable drug-likeness characteristics and enhanced potential for oral bioavailability. These positive outcomes suggest that these compounds exhibit properties that align with established criteria for drug repurposing. Aripiprazole had some advantageous drug-likeness characteristics, but it showed a violation of the Ghose rule for Molar Refractivity (MR > 130) and violations of the Leadlikeness criteria (MW > 350, XLOGP3 > 3.5). Methadone had a bioavailability score of 0.55, indicating a moderate potential for oral bioavailability but it met Lipinski’s “Rule of Five” criteria and is predicted to have good drug-likeness according to other assessments (Ghose, Veber, Egan, Muegge). On the other hand, opioids, rescinnamine, and glyceryl trinitrate had multiple violations and several unfavorable characteristics for oral bioavailability and drug-likeness, which raise concerns about their potential as drug candidates. Pentobarbital appears to have favorable characteristics, but its low molecular weight and potential drug synthesis and formulation challenges may affect its drug likeness and oral bioavailability. Amrubicin also demonstrates favorable attributes but its low GI absorption and potential interactions with P-gp could impact its oral bioavailability. In a similar framework, emetine has certain drug-like attributes, but it also displays some disadvantageous characteristics that hinder its progression as a viable drug candidate.

**Table 2:** Drug-likeness assessment of the potential drug candidate for repurposing from PharmGKB (first 10 rows) and DrugTargetor (last 6 rows).

	Drugs	Lipophilicity consensus log Po/w	Size (MW; gr/mol)	Polarity	Solubility	Flexibility (rotatable bonds)	Saturation	Bioavailability score
**1.**	**Paroxetine**	**3.50**	**329.37**	39.72	**Moderately, moderately, poorly**	**4**	**0.37**	**55% (FDA approved)**
2.	Fluoxetine	4.32	309.33	21.26	Moderately, moderately, poorly	7	0.29	55%
3.	Citalopram	3.68	324.39	36.26	Soluble, soluble, poorly	5	0.35	55%
4.	Venlafaxine	2.21	277.40	32.70	Very, highly, moderately	5	0.65	55%
3	Bupropion	3.15	239.74	29.10	Soluble, soluble, moderately	4	0.46	55%
6.	Risperidone	3.62	410.48	64.16	Moderately, moderately, poorly	4	0.52	55%
7.	Aripiprazole	4.21	448.39	44.81	Moderately, moderately, poorly	7	0.43	55% (1 violation)
8.	Quetiapine	2.77	383.51	73.60	Soluble, soluble, moderately	6	0.38	55%
9.	Olanzapine	2.91	312.43	59.11	Soluble, soluble, moderately	1	0.35	55%
10.	Clozapine	2.98	326.82	30.87	Moderately, soluble, poorly	1	0.28	55%
11.	Amrubicin	0.82	483.47	176.61	Soluble, moderately, soluble	3	0.40	55% (Orphan Drug)
12.	Methadone	4.06	309.45	20.31	Moderately, moderately, poorly	7	0.38	55%
13.	Emetine	4.24	480.64	52.19	Moderately, moderately, Poorly	7	0.59	55%
14.	Glyceryl trinitrate	−1.68	227.09	165.15	Very, moderately, soluble	8	1.00	55%
15.	Rescinnamine	3.91	634.72	117.78	Poorly, poorly, poorly	11	0.49	17%
16.	Prenylamine	5.39	329.48	12.03	Moderately, moderately, poorly	8	0.25	55% (withdrawn)

The second column shows the drug examined, the pharmacokinetic profile of these drugs and the 6 specific characteristics (lipophilicity, size, polarity, solubility, flexibility, and saturation) are shown in the third till eight column and the bioavailability score of said drugs in the ninth column. In regard to the table as a whole: green shows that the value of the characteristic studied is within the optimal threshold; yellow shows that the value of the characteristic studied is within the threshold but not toward the optimal value; red shows that the value of the characteristic studied is not within the threshold. In regard to the second column: the first drug (in green and bold) is the drug that has also been FDA approved for PTSD; the drugs from row 2 to 9 are FDA approved drugs used as off-label treatment for PTSD; clozapine which had a quite favorable pharmacokinetic profile but has not been extensively linked with PTSD; drugs in row 10 and 11 have not been previously linked with PTSD; the drugs from row 12–16 (in red) are drugs that did not score very well in their pharmacokinetic profile. Rescinnamine had the lowest bioavailability score of 17% and Prenylamine is withdrawn in some countries.

### 3.5 Mathematical modeling for drug repurposing

The Lotka-Volterra model describes the dynamics of two populations interacting under a predator–prey basis. The model is adjusted to interpret one population as representing individuals with PTSD and another population as representing individuals without PTSD. The model dynamics will be interpreted as the interplay between these populations in the context of PTSD development. Therefore, to reframe the model in regard to PTSD, $u\left(t\right)$ represents the population of individuals with PTSD at time t and $v\left(t\right)$ represent the population of individuals without PTSD at time t . The modified model equations become:


$$\frac{du}{dt}=cu+b_{u}D_{u\left(t\right)}u$$

$$\frac{dv}{dt}=cv+b_{v}D_{v\left(t\right)}v$$

Where, $b_{u}$ represents the rate of change of the activity of individuals with PTSD due to the drug effect, and $b_{v}$ represents the rate of change of the activity of individuals without PTSD due to the drug effect. $D_{u\left(t\right)}$ and $D_{v\left(t\right)}$ represent the drug doses administered to individuals with and without PTSD, respectively, at time *t*. The drug doses depend on the specific drug being repurposed and the dosing schedule. The terms $b_{u}D_{u\left(t\right)}u$ and $b_{v}D_{v\left(t\right)}v$ represent the drug repurposing effects on the populations. The values of $b_{u}$ and $b_{v}$ can be positive or negative, depending on whether the drug has a beneficial or detrimental effect on individuals with and without PTSD. c is a constant representing the specific rate of growth or decay (mortality) of individuals with and without PTSD.

## 4 Discussion

The main findings of this work can be divided into three main axes. The first axis focuses on compiling all the genes (122 genes) with genetic variations (184 SNPs) that are associated with PTSD. This represents the main step towards proposing personalized pharmacological approaches. The second step is aligned with the second axis which focused on identifying the pathways that are associated with these genes. The final axis focused on analyzing the pharmacokinetic profiles of the potential drug candidates identified as well as proposing a mathematical model for drug repurposing. The findings indicated that the pharmacokinetic profiles of the drugs used as off-label treatment for PTSD were favorable. The drugs identified using DrugTargetor did not have promising profile. Two compounds clozapine, and amrubicin had promising characteristics.

The gene ontology enrichment analysis for the gene targets of the top 17 drug compounds showed certain enriched terms such as neurotransmitter receptor activity and serotonin binding findings that align with existing knowledge of neurotransmitter dysregulation in PTSD. Other terms involve processes that play a role in physiological mechanisms as well as disturbances that may play a role in the disrupted neural circuits associated with fear learning and extinction, which are also key processes in PTSD (Luchkina and Bolshakov, 2019). Additional, enriched categories showed contributions to the communication between nerve cells and the regulation of blood flow, essential factors in the context of PTSD. Other terms suggest their involvement in diverse metabolic activities and rhythmic patterns, potentially offering insights into the regulatory processes that influence PTSD-related physiological responses. The enrichment of the cellular components ‘neuron projection’ and ‘cytoplasmic vesicles’ suggests that the genes involved in these categories may impact neuronal connectivity and communication, which are crucial for understanding how traumatic experiences can lead to persistent changes in neural circuits associated with fear and emotional regulation (Fenster et al., 2018). These persistent changes are one of the hallmarks of PTSD. The KEGG enrichment analysis showed that the folate biosynthesis pathway was enriched, which highlights the importance of folate in supporting various physiological functions, including DNA methylation and neurotransmitter synthesis (Lintas, 2019). Disruptions in folate metabolism have been linked to altered neural plasticity and mood regulation (Falade et al., 2021), potential contributing factors to the emotional dysregulation and cognitive impairments observed in PTSD. The pathways associated with cocaine addiction and alcoholism emphasize the intricate relationship between substance use disorders and PTSD which is further examined below (Flanagan et al., 2016). The enrichment of the neurotransmitter signaling pathways aligns with the well-established role of neurotransmitters in regulating mood, cognition, and stress responses (Jiang et al., 2022), and dysregulation of these pathways can be linked to the emotional and cognitive disturbances seen in PTSD. The enrichment seen in the Cellular Communication and Signaling pathways points out the importance of intercellular communication and signaling in neural circuits. Dysfunctions in these processes can disrupt information flow between brain regions, potentially contributing to cognitive deficits and altered emotional processing which are observed in PTSD (Girgenti et al., 2017). Additionally, dysregulation in the metabolism and hormonal levels can impact stress responses, contributing thus, to the development of PTSD symptoms (Dell’Oste et al., 2023).

The drug repurposing methodology identified specific drugs that exhibited statistically significant enrichment for their associations with the 122 genes linked to PTSD. These drugs include antidepressants; venlafaxine, bupropion, and SSRIs; fluoxetine, citalopram, paroxetine, and opioids; methadone, and antipsychotics; olanzapine, risperidone, aripiprazole, clozapine, and quetiapine (Table 1). The aforementioned compounds can be categorized into antipsychotics, antidepressants, and opioids.

SSRIs are a type of antidepressant medication that inhibits serotonin’s reabsorption. This mechanism enables an amount of the neurotransmitter to remain available in the synaptic clefts leading to increased levels in the brain. Sertraline is one of the most extensively studied SSRIs in regard to PTSD (Huang et al., 2020) and although it was not included in the top 15 most promiscuous drugs as shown in the results of our analysis, sertraline was part of the top 20 drug compounds. Research shows that Sertraline has a positive effect in reducing certain PTSD symptoms such as re-experiencing, avoidance, and hyperarousal (Graham et al., 2020). Using DrugBank^12^ it was also discovered that Sertraline has been extensively studied in 25 PTSD-related clinical trials. A common point of interest in many of these trials was the side effects, including gastrointestinal disturbances, dizziness, and sleep disturbances, noted as a result of the Sertraline treatment (Alexander, 2012). Research, as well as clinical trials (21 relevant trials were identified using DrugBank), showed that Paroxetine, another SSRI, leads to improved quality of life for individuals with PTSD (Huang et al., 2020) and reduced PTSD symptoms, particularly in combat-related cases. Similar were the finding for the SSRI, Fluoxetine. Additionally, DrugBank also linked the SSRI Citalopram (mostly used for depression treatment) with PTSD. However, it is not as associated with PTSD as other SSRIs. In addition to SSRIs, the SNRI Venlafaxine has also shown promise in treating PTSD symptoms (Sonne et al., 2016). Bupropion, which was shown to target 12 out of the 122 identified genes associated with PTSD, has not been concretely shown to be an effective treatment. Trials involving treatment with Bupropion in PTSD, once again identified using DrugBank, did not show significant evidence of its effectiveness (Patel et al., 2016) but there have been some findings that showed potential benefits of Bupropion for younger patients.

Antipsychotics are compounds that are often prescribed as treatment for mental disorders. As shown in the pharmacogenetics analysis, antipsychotics were among the top 15 drugs identified to target the PTSD-related gene set, both as a broad category but also specifically via olanzapine, risperidone, aripiprazole, clozapine, and quetiapine. Quetiapine and olanzapine were also included in the DrugBank in association with PTSD. Research shows that treatment with quetiapine, which is commonly used to treat bipolar disorder, schizophrenia, and major depressive disorder, can improve the following PTSD symptoms: re-experiencing, avoidance, hyperarousal, flashbacks, depressive symptoms, anxiety, psychotic symptoms, insomnia, and nightmares (Crapanzano et al., 2023). Furthermore, olanzapine, commonly used to manage schizophrenia, bipolar 1 disorder, and agitation associated with these conditions, has been shown to alleviate anxiety- and memory-related behavioral symptoms caused by PTSD in rat models (Reddy and Krishnamurthy, 2018). Risperidone, a second-generation antipsychotic medication was shown to target 15 genes out of the 122 (Table 1). Through the DrugBank, it was identified that risperidone was utilized in a PTSD-related clinical trial that reached phase 4, which aimed to evaluate its clinical response in women with PTSD resulting from domestic violence or rape trauma.

In addition, aripiprazole was shown to target 10 genes from the PTSD-related gene set and is sometimes prescribed as an adjunctive treatment in individuals with PTSD who have not responded well to other treatments. It affects the activity of neurotransmitters in the brain, particularly dopamine and serotonin (de Bartolomeis et al., 2015), leading to significant improvements in primary outcome measures as indicated by CAPS scores or PTSD Checklist-Military (Britnell et al., 2017). Although it’s generally well tolerated some patients may experience treatment-related side effects such as anxiety or insomnia. Clozapine, an antipsychotic primarily used for treating schizophrenia has also been identified as one of the compounds that target genes from the PTSD-related gene set. While it’s not typically the first-line treatment for PTSD there have been promising findings regarding its role in managing severe or treatment-resistant symptoms of PTSD (Abejuela et al., 2014).

The analysis of gene-drug interactions also revealed opioids as statistically significantly enriched. Opioids are commonly used for managing pain and have been linked to 14 genes (Table 1). Opioids, which include both endogenous, opioids produced in the body, and prescription pain medications known as exogenous opioids play a role in regulating stress responses. These findings align with the growing understanding of the effects of opioids including their influence on neurotransmission by affecting stress-related neurotransmitters, like norepinephrine, serotonin, and CRH (Reeves et al., 2022). Additionally, opioids impact response (Liang et al., 2016) and pathways related to addiction (Kudla and Przewlocki, 2021). It is crucial to take this into consideration since there is a problem of opioid misuse. Understanding these interactions can offer insights for improving pain management and tackling the challenges related to opioid misuse. Opioids have both pain-relieving and anxiety-reducing effects, which can temporarily alleviate stress. However, several studies suggest that administering opioids during acute trauma care may potentially lower the likelihood of developing PTSD in some cases. Specifically, one study demonstrated that morphine was linked to a decrease in the risk of PTSD, among injured personnel (Holbrook et al., 2010). The problem with misuse arises because people with PTSD may experience noradrenergic overstimulation, which can make them more prone to self-medicate using substances like alcohol, opioids, and benzodiazepines that have sedative effects (López-Martínez et al., 2019). These substances are used to alleviate symptoms and emotional distress associated with PTSD. They are more of a coping mechanism, than a treatment approach (Dassieu et al., 2019). In this context methadone, an agonist medication is primarily used for treating opioid dependence. Methadone is known to help mitigate withdrawal symptoms in those with opioid use disorder (OUD) (Weiss et al., 2019; Leconte et al., 2022). Although it is not typically the choice of treatment for PTSD there have been studies exploring its potential in managing symptoms associated with the condition. Relevant research conducted on rodents has indicated that irregular dopamine signals in the cortex could play a role in the connection between memories and heightened responsiveness to the rewarding effects of drugs, such as opioids (Jing Li et al., 2018). This increased sensitivity might make individuals with PTSD more susceptible to addiction.

The Disease-based Drug-Target Network further enhances our understanding of the genetic influences on drug targeting. Among the drugs investigated, Rescinnamine, Pentobarbital, Amrubicin, Prenylamine, Emetine, and Gluceryl Trinitrate emerged as key drugs with particularly high −log10(p) values, indicating strong binding affinity to their respective target genes. Rescinnamine was shown to act as an antagonist or negative modulator to SLC18A2. This interaction implies that Rescinnamine may inhibit the activity of SLC18A2, potentially affecting the release of neurotransmitters such as dopamine and serotonin in the nervous system. However, the drug-likeness assessment yielded negative results for the use of rescinnamine as a potential drug candidate for repurposing. Pentobarbital, on the other hand, acts as an agonist or positive modulator for multiple GABA receptor subunits, including GABRG3, GABRG5, GABRG2, GABRA2, GABRA1, GABRB2, GABRD, and GABRA4. As a positive modulator, Pentobarbital may enhance the activity of GABA receptors, leading to increased inhibitory neurotransmission in the brain. This increased inhibition results in a calming and sedative effect on the brain and can lead to a reduction in anxiety, agitation, and other symptoms associated with hyperactivity in the brain. Due to its ability to enhance GABAergic neurotransmission, pentobarbital has been used in the past as a sedative, anesthetic, and anticonvulsant (Fang et al., 2018; Xu et al., 2021). However, its use has significantly declined in recent years due to safety concerns, including the risk of overdose, dependence, and the potential for abuse which is also aligned with the drug-likeness assessment results. Amrubicin was found to target TOP2A as an antagonist or negative modulator. TOP2A is an enzyme involved in DNA replication and repair, and its inhibition by Amrubicin may interfere with cell division and growth, making it an important target in cancer treatment but has not been used for the management of mental health conditions such as PTSD (Jalal et al., 2017; Uusküla-Reimand and Wilson, 2022). Prenylamine was withdrawn from the Canadian, US, and UK markets in 1988 due to concerns regarding cardiac arrhythmias (Shah and Stonier, 2018) which corresponds to the negative drug-likeness assessment that it yielded (chart not shown in Figure 6). Emetine targets HIF1A via an unknown mode of action. Emetine is a natural alkaloid and has been extensively studied in the context of memory research, particularly memory reconsolidation. It has been used to interfere with reconsolidation processes in animal models (Wang, 2022). Studies showed that intra-hippocampal infusion of emetine after memory reactivation has been shown to impair the reconsolidation of certain memories. It disrupts the process by which memories are stabilized after retrieval, leading to memory impairment (Correa et al., 2022). Emetine’s ability to interfere with memory reconsolidation raises potential therapeutic applications in memory-related disorders. It could be explored as a tool to disrupt maladaptive memories, such as those associated with traumatic events. However, its moderate-to-negative drug-likeness assessment may hinder it from being a valuable drug candidate for repurposing. Glyceryl Trinitrate (Nitroglycerin) targets GUCY1A2 as an agonist/positive modulator. A study showed that Veterans with PTSD were more often prescribed long-acting Glyceryl Trinitrate compared to those without PTSD (39% vs. 14%) (Parikh et al., 2019). Glyceryl Trinitrate is a medication commonly used to treat angina that occurs as a result of reduced blood flow to the heart and is not a primary or standard treatment for PTSD.

While these computational assessments show the potential oral bioavailability and drug-likeness assessment, further experimental studies would be needed to confirm their actual behavior *in vivo*, in order to be considered as potential candidates for drug-based therapeutic interventions in PTSD. Many of them show highly promising potential to be repurposed as gene-specific targets that contribute to more efficient and personalized PTSD treatments. By identifying specific genetic variations in PTSD patients and through the aforementioned work to be able to link said genes with certain dysregulate pathways, the pharmacological approach to be followed will have a personalized aspect. More in particular, if the variation is in the genes HTR2A or SLC6A4 which are genes related to serotonin production, the proposed drugs would be SSRIs.

## 5 Conclusion

PTSD is a condition that is influenced by multiple factors and when it comes to treatment strategies, it’s important to consider individual differences and safety profiles. To address this, an integrated approach that combines genetics, computational pharmacology, and mathematics was developed. The goal was to identify treatment strategies for PTSD and provide a mathematically informed prediction process. Gene targets were identified, and drug-target interactions were studied in order to identify potential drug candidates for repurposing. Analyses including gene ontology enrichment analysis, KEGG pathway enrichment, and PPI network analysis were conducted to better understand PTSD-related pathways. The pharmacogenetics approach followed in this work was mostly gene-driven, and future work will focus on the pathways identified. In general, the study on pharmacogenetics has shown results in the field of repurposing drugs for PTSD. Most of the identified drugs have already been used as off-label treatment for PTSD, and the analysis showed that they have favorable pharmacokinetic profiles. Two compounds, clozapine, and amrubicin, also had promising profiles with clozapine being previously linked with decreased psychiatric symptoms and amrubicin, which is mostly related to cancer treatment, having shown no PTSD-association. However, it is important to validate and replicate these findings in large cohorts to establish the clinical relevance of the associations, between drugs and genes that were identified in this research.

## Data availability statement

The original contributions presented in the study are included in the article/supplementary material, further inquiries can be directed to the corresponding author.

## Author contributions

KS: Writing – original draft, Conceptualization, Methodology, Visualization. PV: Supervision, Writing – review & editing, Methodology, Validation.

## Footnotes

^1^ https://www.disgenet.org/

^2^ https://www.ebi.ac.uk/gwas/

^3^ https://www.pharmgkb.org/

^4^ https://drugtargetor.com/

^5^ https://biit.cs.ut.ee/gprofiler/gost

^6^ https://genecodis.genyo.es/

^7^ https://www.r-project.org/

^8^ http://bioinformatics.sdstate.edu/go51/

^9^ https://string-db.org/

^10^ https://metascape.org/gp/index.html#/main/step1

^11^ http://www.swissadme.ch

^12^ https://go.drugbank.com/
